# Factors associated with changing alcohol consumption during the first UK lockdown

**DOI:** 10.1093/eurpub/ckac124

**Published:** 2022-09-12

**Authors:** Kareena McAloney-Kocaman, Kerri E McPherson, Emily McGlinchey, Cherie Armour

**Affiliations:** Department of Psychology, Glasgow Caledonian University, Glasgow G4 0BA, UK; Department of Psychology, Glasgow Caledonian University, Glasgow G4 0BA, UK; Stress Trauma and Related Conditions (STARC) Research Lab, School of Psychology, Queen’s University Belfast, Belfast BT9 5BN, UK; Stress Trauma and Related Conditions (STARC) Research Lab, School of Psychology, Queen’s University Belfast, Belfast BT9 5BN, UK

## Abstract

**Background:**

In response to COVID-19 there have been lockdowns and restrictions to hospitality services. Drinking behaviours often change in response to traumatic events and changes in the drinking environment, and this is influenced by a range of factors. This study explores self-reported changes in alcohol consumption in the third month of the UK lockdown, associations with socio-demographics factors and with COVID-19-related concerns, and mental health and wellbeing.

**Methods:**

The COVID-19 Psychological Wellbeing Study was a longitudinal, online, three-wave survey of 1958 UK adults. Data were collected during the first UK lockdown; wave 1 launched 23 March 2020, wave 2 was 1 month after and wave 3 2 months after completion of wave 1A hierarchical multinomial regression model was estimated to investigate factors associated with changes in perceived alcohol consumption in the third month of the lockdown.

**Results:**

The majority of participants reported changes in drinking (62%) with over one-third indicating increased consumption. Student status and worries about the financial implications of COVID-19 were associated with lower odds of decreased alcohol consumption. Those with above average income and those with children in the household had lower odds of increased alcohol consumption, while younger adults had higher odds of increased alcohol consumption.

**Conclusions:**

This study adds to the growing body of research showing changes in alcohol consumption behaviours during the COVID-19 lockdown restrictions, and identifies risk and protective factors which can aid in targeting intervention at those most in need of support.

## Introduction

A key intervention in the response to the COVID-19 pandemic has been ‘lockdowns’, where the public were required to stay at home, except in specific circumstances such as employment as a key worker, medical emergencies, essential shopping and taking exercise. In the UK there have been restrictions, at various levels, on social mixing since 23 March 2020, with most coming to an end in August 2021. During this time, there have also been periods of closure, restricted opening hours and space use for public houses and licenced premises. Alcohol off sales and consumption in private premises became the predominant drinking context for much of the pandemic.^1^

Alcohol consumption is a modifiable health behaviour and one of the leading causes of morbidity and mortality globally, leading to 3 million deaths per year.^2^ Within the UK, alcohol-related harm presents a substantial burden to the National Health Service (NHS) and the wider economy, in addition to the human cost of disability, illness and death.^3^ As such, understanding patterns of alcohol consumption, and the factors associated with consumption, is critical to support public health measures.

Alcohol consumption is known to vary by both individual (e.g. age, gender, socio-economic status) and societal factors (e.g. drinking culture, context and regulation). The shift to lockdown and social mixing restrictions presented a significant change in societal context, and one we need to better understand the implications of. For example, Rehm *et al*.^4^ posit that alcohol consumption in the aftermath of the COVID lockdowns was likely to decrease in the short-term due to lack of availability and cost, but increase in the medium and long-term in part due to psychological distress experienced during the pandemic, particularly among men.

Emerging evidence suggests that, as in previous pandemics and crisis events,^5^ there has been a trend towards greater alcohol consumption at a population level during the initial stages of COVID-19, although over the longer-term consumption may decrease.^6–8^ Alcohol sales at the start of lockdown restrictions in Germany were 6.1% higher than the same weeks the previous year,^9^ and increased by 67% in the UK.^10^ Early changes in consumption have been noted in China,^11^ Australia,^12^ Germany,^9^ Poland,^13^ the USA^14^ and the UK^15–17^; however, the pattern of change has been mixed with both decreases and increases reported. For example, in Poland 16% of people reported drinking less, while 14% reported drinking more.^13^ Similarly, in a study of French speaking adults in Belgium, France and Canada, 24.5% reported decreased alcohol comparison and 26.4% reported increased consumption during lockdown.^18^ In a UK study, 17% self-reported increased alcohol consumption during the first lockdown.^16^

Even where population level stability has been reported, at an individual level factors such as age, gender and stress have been identified as key vulnerabilities for increased alcohol consumption during lockdown.^19^^,^^20^ For example, in Australia younger individuals were more likely to report decreased consumption, while middle aged females and those experiencing greater stress were more likely to report increases.^19^^,^^20^ Indeed, these factors have been identified in much of the existing research, with increased drinking during lockdown associated with older age,^13^^,^^17^^,^^18^ being female,^17^ higher education,^9^^,^^17^ higher stress level,^9^^,^^18^ poorer mental health^12^^,^^16^ and having children.^12^^,^^18^ Depression, anxiety and PTSD have been identified in mediating the association between exposure to crisis events, such as pandemics, and alcohol consumption.^5^

While evidence is rapidly emerging, there is limited research on patterns of alcohol consumption during COVID-19 restrictions. Of the existing research much is cross-sectional in nature, longitudinal data is often secondary data analysis of sources where data collection tools are not designed to fully capture the COVID-19 circumstances,^15^^,^^17^ or primary data collection studies from countries other than the UK, the findings of which may not be transferable to the UK context. While the COVID-19 pandemic has impacted globally, it is important to understand how restrictions at a country level impact on those specific populations. Different countries and population share different relationships with alcohol so the psychosocial context is important. This paper reports on the COVID-19 Psychological Wellbeing Study (CVPWS), a multi-wave, longitudinal survey of adults in the UK during the first 12 weeks of the first UK lockdown.^21^ This analysis explores self-reported changes in alcohol consumption in the third month of the lockdown and investigates associations with socio-demographics factors and longitudinal associations with COVID-19-related experiences and concerns, and mental health and wellbeing in the first 2 months.

## Methods

Full details of the CVPWS are available in a methodology overview paper,^21^ what is described here are the details that are pertinent to this analysis.

### Study design

The CVPWS was a rapid response, longitudinal, multi-wave online survey, hosted on *Qualtrics*. Wave 1 launched on 23 March 2020, when the UK Government announced a national lockdown from 26 March and it closed on 25 June 2020. Wave 1 data collection was undertaken in the first month of the lockdown, wave 2 in the second month of lockdown 1 month after completion of wave 1 and wave 3 in the third month 2 months after completion of wave 1. The study was approved by Faculty of Engineering and Physical Sciences Ethical Committee at Queen’s University Belfast (EPS 20_96) and Glasgow Caledonian University’s Health and Life Sciences Ethics Committee (HLS/PSWAHS/19/157).

### Recruitment and procedure

Participants were recruited via a social media campaign (e.g. Facebook and Twitter) and the online participant panel *Prolific*. *Prolific* panel members produce high-quality data, and tend to be more diverse and naive to survey completion in comparison to members of other online participant panels.^22^ Eligible participants had to be 18 years or older, resident in the UK at the time of completion, and have a level of English that allowed for unaided participation. Participants recruited via *Prolific* were compensated for their time (£1–2) and participants recruited via social media were entered into a prize draw for one of six £150 vouchers. Wave 1 participants were contacted by email to complete waves 2 and 3. Wave 1 participants who did not complete wave 2 but who did not actively withdraw were contacted to complete wave 3. The consent process was completed at each wave.

### Measures

Socio-demographic characteristics were reported at baseline, alcohol consumption at wave 3 follow-up, and all other items included in this analysis collected at baseline and wave 2.

#### Socio-demographics

Participants were asked to provide information about country of residence, gender, age, relationship status, living arrangements (other adults in the household, children under 18 in household, pet ownership), education, employment status, whether or not they were a key worker, ability to work at home during the pandemic, whether or not they were enrolled as a student, rurality, perceived income (average, below and above average), current and previous self-reported physical and mental health. In each wave, COVID-19-related worry items assessed participants’ level of worry about quarantine/self-isolation, infection concern, stigmatization due to exposure, financial implications, food shortages, the government’s and healthcare systems ability to manage the outbreak, and border closures.

##### Mental health and wellbeing

Anxiety was assessed using the seven-item Generalized Anxiety Disorder scale (GAD-7).^23^ GAD-7 scores range from 0 to 21, with higher total scores reflecting greater severity. The GAD-7 has been reported as a valid and reliable measure of anxiety.^23^

Depression was assessed using the nine-item Patient Health Questionnaire (PHQ-9).^24^ Scores range from 0 to 27, with higher scores indicating higher levels of depressive symptomology. The PHQ-9 has demonstrated excellent reliability.^24^

Loneliness was assessed using the UCLA three Item Loneliness Scale.^25^ Higher scores reflect higher levels of loneliness, and the measure has demonstrated acceptable reliability given the low number of items.^25^

Perceived social support was assessed using the six item Perceived Social Support Questionnaire–Brief Form, which has demonstrated excellent psychometric properties.^26^ Higher scores reflect higher levels of perceived social support.

The presence and pursuit of meaning in life was assessed using the 10-item Meaning in Life Questionnaire (MLQ).^27^ Higher scores on the sub-scales represent increased presence of meaning in life and more active seeking of the meaning or purpose in the respondent’s life. The MLQ has been reported as psychometrically sound.^28^

##### Changes in alcohol consumption

In wave 3, participants were asked to indicate whether, since the introduction of the UK Government lockdown, they believed they had been drinking less, about the same, or more than usual.

### Data analysis

Data were screened and cases removed prior to analysis if the respondent: did not provide data allowing assessment against the inclusion criteria, or they did not meet these; did not complete any of the survey items; or, completed the survey in less than the minimum time. Additionally, data were restricted to individuals who had completed the alcohol consumption item at wave 3.

Descriptive and univariate analyses conducted in SPSS v25 explored the proportions reporting changes in alcohol consumption at wave 3, and associations with socio-demographics, and wave 1 and 2 COVID-19-related worries, health and wellbeing. Hierarchical multinomial regression models were estimated in Mplus Version 8.0 for wave 3 changes in reported alcohol use with ‘drinking the same as usual’ as the reference class. Variables identified as significantly associated with changes in alcohol consumption in the univariate analyses were entered as independent variables in regression models. Models were estimated in a first step of demographic predictors, step two with demographics and wave 1 COVID-19-related worries and mental health variables, and step three with demographics and wave 2 worries and mental health variables.

## Results

The survey was completed by 1958 individuals (29.5% male) at baseline, 85% (*n* = 1660) went on to complete wave 2 and 80% (*n* = 1573) completed wave 3. Participant characteristics of the full sample at wave 1 are reported elsewhere.^17^ Analysis was restricted to the 1268 individuals who completed the alcohol consumption items at wave 3. The majority were female (70.1%) and aged between 18 and 84 years (*M* = 35.06, SD = 13.04). Socio-demographic characteristics are reported in table 1. Examination of attrition between wave 1 and those with data on the wave 3 alcohol consumption item indicated that those who did not complete the wave 3 item had significantly higher levels of wave 1 anxiety and depression, higher worries about being stigmatised and food shortages, but lower levels of perceived social support and lower levels of searching for meaning of life. Younger people, unemployed people, students, those who perceived their incomes to be below average, and those with a pre-existing mental health condition were more likely to complete baseline measures only, and thus be excluded from this analysis. (Supplementary data S1).

**Table 1:** Sample characteristics (*N* = 1268)

Variable	*N*	%
Gender		
Male	372	29.3
Female	889	70.1
Missing	7	0.6
Age		
18–24	188	14.8
25–34	406	32.0
35–44	308	24.3
45–54	199	15.7
55–64	123	9.7
65 and over	44	3.5
Country of residence		
England	509	40.1
Northern Ireland	280	22.1
Scotland	445	35.1
Wales	34	2.7
Employment		
Employed	938	74.0
Not employed	330	26.0
Education level		
Secondary or less	323	25.5
Post-secondary up to degree	618	48.7
Postgraduate	317	25.0
Missing	10	0.8
Student status		
Student	162	12.8
Not a student	1106	87.2
Keyworker status		
Keyworker	488	38.5
Not a keyworker	780	61.5
Adults in household		
Lone adult	248	19.6
Other adults	1020	80.4
Children in household		
Children	468	36.9
No children	800	63.1
Ability to WAH		
Able to WAH	887	70.0
Not able to WAH	381	30.0
Urbanicity		
Rural	274	21.6
Town	573	45.2
City	421	33.2
Perceived income		
Average or below	1037	81.8
Above average	231	18.2
Pet ownership		
Owns a pet	673	53.1
No pet	595	46.9
Physical health		
Has a physical health condition	321	25.3
No physical health condition	947	74.7
Mental health		
Has a mental health condition	367	28.9
No mental health condition	901	71.1

At wave 3, 38.1% of the sample reported drinking the same as usual, 35.7% an increase in alcohol consumption and 26.2% a decrease. As can be seen in table 2, perceived changes in alcohol consumption were significantly associated with age, employment status, employment as a key worker, ability to work at home, presence of children in the household, being enrolled as a student, pet ownership, perceived income, wave 1 COVID-19-related worries about quarantine, financial implications, food shortages, UK Government’s ability to manage the pandemic, health service ability to cope and the impact of border closures, wave 1 loneliness, anxiety, depression, and both presence and search of meaning in life, and wave 2 COVID-19-related worries about quarantine, financial implications, food shortages, UK Government’s ability to manage the pandemic and health service ability to cope with patients, anxiety, depression and search for meaning in life. *Post hoc* tests are available on request from the authors, but are not presented here as univariate tests were used solely for item reduction purposes.

**Table 2:** Self-reported changes in alcohol consumption at wave 3 by demographics, wave 1 and wave 2 measures

Variable	Alcohol consumption in wave 3 (month 3 of lockdown)			
	Less than usual	Same as usual	More than usual	Inferential test result
Demographics	%	%	%	
Gender				
Male	26.3	40.1	33.6	χ^2^_(2)_ = 1.208, *P* = 0.547
Female	26.0	37.3	36.7	
Age				χ^2^_(10)_ = 91.644, *P* < 0.001
18–24	51.1	23.9	25.0	
25–34	25.6	40.9	33.5	
25–44	19.8	36.4	43.8	
45–54	21.1	37.2	41.7	
55–64	15.4	51.2	33.3	
65 and over	22.7	52.3	25.0	
Lone adult in house	26.6	40.3	33.1	χ^2^_(2)_ = 1.036, *P* = 0.596
Not lone adult	26.1	37.5	36.4	
Key worker	21.9	37.9	40.2	χ^2^_(2)_ = 9.871, *P* = 0.007
Not a key worker	28.8	38.2	32.9	
Able to WAH	28.1	36.4	35.5	χ^2^_(2)_ = 6.204, *P* = 0.044
Not able to WAH	21.8	42.0	36.2	
Employed	23.7	38.2	38.2	χ^2^_(2)_ = 14.726 *P* = 0.001
Not employed	33.3	37.9	28.8	
Children in household	19.9	37.8	42.3	χ^2^_(2)_ = 20.294 *P* < 0.001
No children in household	29.9	38.3	31.9	
Pre-existing physical health condition	23.7	38.0	38.3	χ^2^_(2)_ = 1.841, *P* = 0.398
No pre-existing physical health condition	27.0	38.1	.34.8	
Pre-existing mental health condition	30.8	37.1	32.2	χ^2^_(2)_ = 6.178, *P* = 0.0.46
No pre-existing mental health condition	24.3	38.5	37.2	
Education level				
Full secondary education or less	28.8	39.6	31.6	χ^2^_(4)_ = 6.149, *P* = 0.188
Undergraduate	24.8	39.3	35.9	
Postgraduate	26.8	33.8	39.4	
Student	44.4	22.8	32.7	χ^2^_(2)_ = 35.469, *P* < 0.001
Not a student	23.5	40.3	36.2	
Owns a pet	23.0	39.5	37.4	χ^2^_(2)_ = 1.036, *P* = 0.025
Does not own a pet	29.7	36.5	33.8	
Urbanicity				χ^2^_(4)_ = 6.852, *P* = 0.144
Rural	25.9	38.7	35.4	
Town	24.6	41.4	34.0	
City	28.5	33.3	38.2	
Income				χ^2^_(2)_ = 16.827, *P* < 0.001
Less than average	16.9	37.7	45.5	
Average or above	38.1	28.3	33.3	
Wave 1 (month 1) Measures	M (SD)	M (SD)	M (SD)	
Worries				
About quarantine/self-isolation	2.36 (1.06)	2.25(1.06)	2.49 (1.08)	F_(2, 1264)_ = 5.525, *P* = 0.004
About being infected	2.98 (1.17)	3.07 (1.16)	3.15 (1.1.5)	F_(2, 1264)_ = 1.928, *P* = 0.146
About infecting others	3.52 (1.18)	3.46 (1.18)	3.64 (1.10)	F_(2, 1264)_ = 2.810, *P* = 0.061
About being stigmatized	1.85 (1/08)	1.72 (1.08)	1.85 (1.13)	F_(2, 1264)_ = 2.060, *P* = 0.129
About financial implications	3.34 (1.26)	3.22 (1.29)	3.57 (1.28)	F_(2, 1264)_ = 9.173, *P* < 0.001
About food shortages	2.63 (1.15)	2.53 (1.13)	2.74 (1.15)	F_(2, 1264)_ = 3.832, *P* = 0.022
About UK government ability	3.45 (1.19)	3.24 (1.15)	3.46 (1.20)	F_(2, 1264)_ = 5.500, *P* = 0.004
About Health service ability to cope	3.91 (1.03)	3.80 (1.05)	4.05 (0.99)	F_(2, 1264)_ = 6.821, *P* = 0.001
About impact of border closures	2.32 (1.29)	2.10 (1.15)	2.34 (1.29)	F_(2, 1264)_ = 5.210, *P* = 0.006
Loneliness	5.45 (1.86)	5.14 (1.92)	5.43 (1.93)	F_(2, 1264)_ = 3.687, *P* = 0.025
Social support	21.71 (5.61)	21.58 (5.74)	22.28 (5.60)	F_(2, 1264)_ = 1.978, *P* = 0.139
Anxiety	7.36 (6.12)	6.15 (5.64)	7.89 (5.89)	F_(2, 1264)_ = 10.790, *P* < 0.001
Depression	8.22 (6.46)	6.67 (6.02)	8.13 (6.50)	F_(2, 1264)_ = 8.456, *P* < 0.001
Meaning in life				
Presence	16.40 (5.04)	17.30 (5.48)	17.36 (5.52)	F_(2, 1264)_ = 3.169, *P* = 0.027
Search	22.17 (7.35)	20.70 (7.54)	21.22 (7.90)	F_(2, 1264)_ = 3.652, *P* = 0.026
Wave 2 (Month 2) measures				
Worries				
About quarantine/self-isolation	2.18 (1.05)	1.97 (1.02)	2.15 (1.06)	F_(2, 1198)_ = 4.940, *P* = 0.007
About being infected	2.74 (1.08)	2.74 (1.13)	2.89 (1.13)	F_(2, 1198)_ = 2.421, *P* = 0.089
About infecting others	3.12 (1.17)	3.02 (1.14)	3.08 (0.20)	F_(2, 1198)_ = 0.791, *P* = 0.454
About being stigmatized	1.63 (1.00)	1.63 (0.93)	1.74 (1.09)	F_(2, 1198)_ = 1.535, *P* = 0.216
About financial implications	3.15 (1.24)	2.93 (1.30)	3.30 (1.28)	F_(2, 1198)_ = 9.245, *P* < 0.001
About food shortages	2.02 (0.99)	1.98 (1.03)	2.16 (1.09)	F_(2, 1198)_ = 3.360, *P* = 0.035
About UK government ability	3.26 (1.22)	3.09 (1.19)	3.36 (1.20)	F_(2, 1198)_ = 5.985, *P* = 0.003
About Health service ability to cope	3.22 (1.14)	3.09 (1.09)	3.36 (1.13)	F_(2, 1198)_ = 6.718, *P* = 0.001
Loneliness	5.49 (1.95)	5.15 (1.96)	5.34 (1.95	F_(2, 1197)_ = 2.895, *P* = 0.051
Social support	21.86 (5.62)	21.92 (5.97)	22.51 (5.85)	F_(2, 1997)_ = 1.531, *P* = 0.217
Anxiety	7.07 (5.46)	6.00 (5.36)	7.21 (5.58)	F_(2, 1197)_ = 6.431, *P* = 0.002
Depression	8.27 (5.93)	6.70 (6.04)	7.97 (6.37)	F_(2, 1197)_ = 7.261, *P* = 0.001
Meaning of life				
Presence	21.89 (7.03)	22.70 (7.83)	23.12 (7.52)	F_(2, 1194)_ = 2.415, *P* = 0.090
Search	7.26 (0.41)	7.42 (0.34)	7.52 (0.36)	F_(2, 1194)_ = 7.251, *P* = 0.001

Hierarchical multinomial regression models of wave 3 changes in alcohol consumption, with same as usual as the reference category, were estimated in three steps (table 3).

**Table 3:** Hierarchical multinomial regression changes in alcohol consumption at wave 3 by demographics and wave 1 and 2 measures

	Less alcohol than usual			More alcohol than usual		
	Step 1	Step 2	Step 3	Step 1	Step 2	Step 3
**Country**						
England (ref)	1.00	1.00	1.00	1.00	1.00	1.00
Northern Ireland	0.68 (0.48–0.97)	0.71 (0.50–1.02)	0.68 (0.47–0.98)	0.72 (0.48–1.07)	0.73 (0.49–1.10)	0.78 (0.51–1.18)
Scotland	0.74 (0.54–1.00)	0.75 (0.55–1.03)	0.78 (0.56–1.08)	0.89 (0.63–1.25)	0.88 (0.62–1.26)	0.92 (0.64–1.33)
Wales	1.21 (0.53–2.80)	1.13 (0.48–2.65)	1.12 (0.46–2.74)	0.95 (0.36–2.53)	0.95 (0.35–2.56)	0.99 (0.35–2.78)
**Age**						
18–24	0.59 (0.24–1.49)	0.82 (0.31–2.17)	0.88 (0.33–2.33)	3.10 (1.11–8.65)	3.67 (1.26–10.75)	3.35 (1.11–10.08)
25–34	0.68 (0.30–1.55)	0.97 (0.41–2.30)	1.00 (0.42–2.37)	1.23 (0.47–3.21)	1.49 (0.55–4.06)	1.50 (0.54–4.19)
35–44	0.48 (0.21–1.12)	0.67 (0.28–1.62)	0.76 0.32–1.82)	0.84 (0.31–2.24)	1.00 (0.36–2.77)	1.03 (0.36–2.93)
45–54	0.49 (0.21–1.16)	0.65 (0.27–1.57)	0.72 (0.30–1.74)	0.90 (0.33–2.46)	1.07 (0.38–2.99)	1.13 (0.39–3.22)
55–64	0.79 (0.34–1.85)	0.99 (0.41–2.38)	1.20 (0.50–2.89)	0.65 (0.23–1.85)	0.72 (0.25–2.08)	0.81 (0.27–2.42)
65 and over (ref)	1.00	1.00	1.00	1.00	1.00	1.00
**Key worker status**						
Not a key worker (ref)	1.00	1.00	1.00	1.00	1.00	1.00
Key worker	0.92 (0.68–1.24)	0.89 (0.66–1.21)	0.85 (0.62–1.16)	0.86 (0.62–1.21)	0.84 (0.60–1.19)	0.83 (0.58–1.19)
**Work at home ability**						
Not able to work at home (ref)	1.00	1.00	1.00	1.00	1.00	1.00
Able to work at home	0.97 (0.72–1.30)	0.92 (0.68–1.25)	0.91 (0.67–1.24)	1.30 (0.91–1.85)	1.27 (0.89–1.81)	1.31 (0.90–1.90)
**Employment**						
Unemployed/Not working (ref)	1.00	1.00	1.00	1.00	1.00	1.00
Employed	0.82 (0.57–1.19)	0.81 (0.55–1.18)	0.79 (0.54–1.17)	0.75 (0.50–1.12)	0.76 (0.50–1.14)	0.68 (0.45–1.05)
**Children in household**						
No children	1.00	1.00	1.00	1.00	1.00	1.00
Children	0.83 (0.63–1.11)	00.84 (0.62–1.14)	0.81 (0.60–1.10)	0.61 (0.44 –0.85)	0.62 (0.44–0.88)	0.65 (0.46–0.92)
**Student status**						
Not a student (ref)	1.00	1.00	1.00	1.00	1.00	1.00
Student	0.54 (0.32–0.93)	0.54 (0.31–0.95)	0.61 (0.34–1.10)	0.73 (0.43–1.24)	0.73 (0.42–1.24)	0.77 (0.44–1.36)
**Pet owner**						
Not a pet owner (ref)	1.00	1.00	1.00	1.00	1.00	1.00
Pet owner	1.05 (0.81–1.38)	1.09 (0.83–1.43)	1.07 (0.81–1.42)	0.75 (0.56–1.02)	0.76 (0.56–1.03)	0.80 (0.58–1.10)
**Income**						
Average or below (ref)	1.00	1.00	1.00	1.00	1.00	1.00
Above average income	0.74 (0.53–1.03)	0.66 (0.46–0.93)	0.67 (0.47–0.96)	0.58 (0.38–0.89)	0.53 (0.35–0.82)	0.53 (0.34–0.84)
**Wave 1 (month 1) Measures**						
**COVID-19-related worries**						
Quarantine		0.93 (0.81–1.08)			0.96 (0.82–1.16)	
Financial implications		0.87 (0.77–0.98)			0.88 (0.76–1.00)	
Food shortages		1.01 (0.88–1.17)			0.97 (0.83–1.14)	
UK Government		0.98 (0.83–1.12)			1.09 (0.92–1.29)	
Health systems		0.94 (0.80–1.12)			0.93 (0.77–1.13)	
Borders		0.95 (0.84–1.07)			1.00 (0.88–1.14)	
**Loneliness**		0.96 (0.88–1.05)			0.95 (0.86–1.05)	
**Anxiety**		0.97 (0.93–1.01)			0.98 (0.93–1.02)	
**Depression**		1.01 (0.97–1.05)			1.02 (0.97–1.06)	
**Meaning of Life**						
Presence		0.99 (0.96–1.02)			0.99 (0.96–1.03)	
Search		1.00 (0.98–1.02)			1.00 (0.98–1.02)	
**Wave 2 (month 2) meaures**						
**COVID-19-related worries**						
Quarantine			0.95 (0.81–1.10)			1.04 (0.88–1.23)
Financial implications			0.93 (0.74–0.95)			0.91 (0.79–1.06)
Food shortages			1.00 (0.85–1.17)			0.93 (0.78–1.12)
UK Government			0.93 (0.80–1.09)			1.01 (0.85–1.20)
Health systems			0.94 (0.80–1.12)			0.91 (0.75–1.10)
**Anxiety**			1.00 (0.96–1.05)			0.99 (0.94–1.04)
**Depression**			0.98 (0.94–1.02)			1.00 (0.96–1.05)
**Meaning of life—search**			1.00 (0.95–1.02)			1.01 (0.99–1.04)

At step 1, participants from Northern Ireland and those who were students had lower odds of decreased alcohol consumption. On inclusion of wave 1 worries and mental health, the association with country of residence became non-significant, but students continued to have lower odds of decreased alcohol consumption, as did those who perceived their income to be above average. Greater wave 1 worries about the financial impact of COVID-19 were also associated with lower odds of decreased alcohol consumption in the third month of the lockdown. In step 3 with removal of wave 1 worries and mental health and inclusion of wave 2 worries and mental health, participants in Northern Ireland again had lower odds of decreased alcohol consumption, as did those who perceived their income to be above average. Greater worry about financial implications of COVID-19 at wave 2 were associated with slightly lower (7%) odds of decreased alcohol consumption.

In step 1, the odds of increased alcohol consumption at wave 3, were significantly lower for those who had children under 18 in the household, and those who perceived their income was above average. Participants aged 18–24 years had over 3 times greater odds of increased alcohol consumption at wave 3. At step 2, no wave 1 worries or mental health variables were significantly associated with increased alcohol consumption. Having children resident in the household and perceiving income to be above average remained associated with lower odds of increased alcohol consumption, and those aged 18–24 years had over 3.5 times higher odds of increased alcohol consumption. Similarly, in step 3, no wave 2 worries or mental health variables were significantly associated with increased alcohol consumption. Having children resident in the household and perceiving income to be above average remained associated with lower odds of increased alcohol consumption, and those aged 18–24 years had over 3 times higher odds of increased alcohol consumption.

## Discussion

Over half the participants believed they had changed their alcohol consumption during the first UK lockdown; over a third reported increased consumption and over a quarter reported decreased consumption. This supports existing research in both the UK and internationally recording changes in alcohol consumption during the COVID-19 pandemic.^11^^,^^12^^,^^14–17^

While several factors were significantly associated with both decreased and increased alcohol consumption in the univariate analyses, the multinomial regression models revealed age, student status, income, worry about finances and children in the household as significantly associated with changed alcohol consumption. Young people aged 18–24 years had substantially higher odds of drinking more alcohol during lockdown in comparison with the oldest group. This is inconsistent with some reports of lockdown drinking where older adults were more likely to increase their drinking behaviours,^13^^,^^17^^,^^18^ but supports other findings that have identified young people as a group particularly at risk of greater alcohol consumption.^29^^,^^30^ Furthermore students had lower odds of reporting decreased alcohol consumption, which contrasts with reports of decreased alcohol consumption by UK students during COVID.^31^ Student status is consistently associated with high alcohol consumption, and often with harmful alcohol use, particularly in the UK.^32^ Here the findings suggest that students have continued to consume alcohol during the pandemic at levels consistent with their prior intake, although it is beyond the scope of this analysis as to whether these levels are harmful or not.

Income was an important factor in changing alcohol consumption, with those reporting above average income less likely to change their consumption, but rather to continue to drink at pre-COVID levels. This may reflect the capacity of these individuals to continue to source alcohol in the same way as pre-pandemic, and therefore not disrupt their consumption habits. There is an acknowledged social gradient in the consumption of alcohol, with middle and higher socio-economic groups associated with regular drinking, facilitated by home and work consumption, and lower socio-economic groups drinking less but more harmfully, including binge drinking.^33^ Therefore, the instigation of lockdowns and restrictions on public alcohol consumption is unlikely to have translated into reduced alcohol-related harm among those from more affluent backgrounds.

Interestingly, those with higher worry about the financial impact of the pandemic in both the first and second months of lockdown also had lower odds of consuming less alcohol. While this may seem counterintuitive, whereby concerns about finances may result in a decreased spend on alcohol, it could highlight the continued use of alcohol as a coping mechanism to deal with worries and concerns, or perhaps that the worries about the financial impacts were focused on future impacts rather than immediate impacts.

Several studies have identified relationships with children as a risk factor for greater alcohol consumption among adults during the lockdown.^12^^,^^18^ However, in this study, the presence of children in the household had a protective role in changes in alcohol consumption. Adults living in household with children present had considerably lower odds of increasing their alcohol consumption. While in contrast to the existing literature, this aligns with other findings from the UK which have also reported better mental health outcomes for those with children in the household.^34^ One possible explanation is that individuals living in households with children did not experience them as a stressor during lockdown, but as a responsibility and source of structure and meaning for their daily lives, and as a result are less likely to engage in alcohol consumption. Moreover, adults typically transition to drinking at home when they become parents,^35^ so it may be that households with children present saw less disruption and change to their drinking behaviour during the lockdowns as it required no change in the context of that drinking.

Interestingly, neither depression nor anxiety emerged as significant predictors in the regression model, despite significant associations at the univariate level. Previous research has linked alcohol consumption with higher rates of negative mental health symptoms, and as playing a mediating role in exposure to traumatic events and depression and anxiety.^5^ The COVID-19 pandemic is an ongoing traumatic event,^34^ and therefore more research is warranted to further understand the inter-relationships between mental health and alcohol consumption as the pandemic progresses.

Gender was not significantly related to changes in alcohol consumption, which contrasts with findings in Australia where females were found to drink more,^19^ and with predictions that males would be most vulnerable to increased alcohol consumption in the aftermath of the pandemic.^4^ Within the UK it appears that both males and females report similar changes in their alcohol consumption patterns. This is not surprising, given that in the three months of this study public houses and licenced premises were not open and alcohol consumption was confined to home environments, with restrictions on visiting anyone outside your own residence. These restrictions impacted equally on both males and females. However, gender differences in alcohol consumption more generally may mean that how males and females perceive changes in their alcohol consumption, and how that relates to actual alcohol consumption and alcohol-related harm may not be captured fully here.^36^^,^^37^

This study provides evidence of the impact of the pandemic from the start of the UK’s first national lockdown, adding to global evidence of how alcohol consumption changed during this time; however, a number of limitations must be noted. The sample was not representative of the UK population, and females in particular are over-represented^21^ therefore the data cannot be claimed to reflect the alcohol consumption behaviours of the UK population. The study was conducted entirely online which may have impacted on the type of participants able to respond, potentially excluding those with limited digital engagement. Additionally, the data were self-report and restricted to perceived changes in alcohol consumption, rather than assessing units of alcohol consumed. Notably due to the responsive nature of the study to the COVID-19 pandemic, measures of pre-lockdown alcohol consumption are not included as a baseline, instead relying on retrospective perceptions of alcohol consumption. However, studies have indicated, that while alcohol consumption self-reports may be vulnerable to response bias, they are a reliable and valid method of assessing alcohol consumption.^38^ Additionally, attrition between wave 1 and follow-up in wave 3 was an issue, with participants with pre-existing mental health conditions, those experiencing higher levels of depression and anxiety at baseline, those with lower social support, younger individuals, and those with lower financial resources less likely to complete the follow-up assessments. Care must be taken in interpreting the results in relation to these groups, particularly where the findings indicate no association with changes in alcohol use, due to this lower representation. Further research should seek to explore the experiences of these groups in particular.

This study demonstrates considerable change in alcohol consumption among UK adults during the COVID-19 lockdown. A range of risk and protective factors for increased alcohol consumption were identified, with young adults being particularly vulnerable to increased consumption. In contrast households with children and above average income emerged as protective factors against increased consumption. This identifies particular groups of individuals who would have benefitted from further support during lockdowns to prevent, or reduce the likelihood, of increased alcohol use. The study also highlights groups of individuals whose alcohol consumption was not influenced or impacted by the lockdown restrictions. It is critical that the alcohol consumption behaviours, and how these change continue to be monitored to understand the impact of COVID-19 more.^39^

## Supplementary data

Supplementary data are available at *EURPUB* online.

*Conflicts of interest*: None declared.

## Data availability

Data requests can be made to the author team.

Key points

- Sixty-two percent perceived a change in their alcohol consumption during the first lockdown.
- Thirty-six percent reported increased alcohol consumption and 26% reported decreased alcohol consumption.
- Students, and those with worries about the financial impact of COVID-19 had lower odds of decreasing their alcohol consumption.
- Younger adults had higher odds of increased alcohol consumption.
- Those with higher incomes, and those with children in the household had lower odds of increased alcohol consumption.

## Supplementary Material

ckac124_Supplementary_Data
