# Supplementary material for: Factors associated with changing alcohol consumption during the first UK lockdown
Source: Eur J Public Health. 2022 Sep 12;32(5):766–72. doi: 10.1093/eurpub/ckac124 (PMC9494400; doi:10.1093/eurpub/ckac124)
Supplement: ckac124_Supplementary_Data [file ckac124_supplementary_data.docx]

Attrition between wave 3 and wave 1 sample by key characteristics

| **Variable** |  |  |  |
| --- | --- | --- | --- |
|  | **Wave 1 only** | **Wave 1 an 3 data** | **Inferential Test result** |
| Demographics | % | % |  |
| Gender |  |  |  |
| Male | 42.8 | 57.2 | χ^2^ _(1)_ = .082, p = .775 |
| Female | 42.0 | 58.0 |  |
| Age |  |  | χ^2^ _(5)_ = 51.574, p <.001 |
| 18 – 24 | 60.2 | 39.8 |  |
| 25 – 34 | 45.0 | 55.0 |  |
| 25 – 44 | 39.9 | 60.1 |  |
| 45 – 54 | 33.8 | 66.2 |  |
| 55 – 64 | 30.7 | 69.3 |  |
| 65 and over | 29.2 | 70.8 |  |
| Employment |  |  | χ^2^ _(1)_ = 12.908 p < .001 |
| Employed | 39.6 | 60.4 |  |
| Not employed | 49.5 | 50.5 |  |
| Education level |  |  | χ^2^ _(2)_ = 4.805, p = .090 |
| Full secondary education or less | 44.6 | 55.4 |  |
| Undergraduate | 39.5 | 60.5 |  |
| Postgraduate | 45.3 | 54.7 |  |
| Student status |  |  | χ^2^ _(1)_ = 21.171, p<.001 |
| Student | 57.0 | 43.0 |  |
| Not a student | 40.7 | 59.9 |  |
| Key worker status |  |  | χ^2^ _(1)_ = 5.282, p = .022 |
| Key worker | 38.6 | 61.4 |  |
| Not a key worker | 44.5 | 55.5 |  |
| Adults in Household |  |  | χ^2^ _(1)_ = .776, p = .378 |
| Lone adult in house | 44.5 | 55.5 |  |
| Not Lone adult | 41.8 | 58.2 |  |
| Children in Household |  |  | χ^2^ _(1)_ = 1.373 p = .241 |
| Children in household | 40.4 | 59.6 |  |
| No children in household | 43.3 | 56.7 |  |
| Ability to WAH |  |  | χ^2^ _(1)_ = 1.192, p = .275 |
| Able to WAH | 43.1 | 56.9 |  |
| Not able to WAH | 40.2 | 59.8 |  |
| Urbanicity |  |  | χ^2^ _(2)_ = 3.354, p = .187 |
| Rural | 42.3 | 57.7 |  |
| Town | 39.9 | 60.1 |  |
| City | 45.1 | 54.9 |  |
| Income |  |  | χ^2^_(1)_ = 6.614, p =.010 |
| Less than average | 46.4 | 53.6 |  |
| Average or above | 39.8 | 60.2 |  |
| Pet Ownership |  |  | χ^2^ _(1)_ = .125, p = .724 |
| Owns a pet | 41.8 | 58.2 |  |
| Does not own a pet | 41.8 | 58.2 |  |
| Physical Health |  |  | χ^2^ _(1)_ = .736, p = .391 |
| Pre-existing Physical health condition | 40.4 | 59.6 |  |
| No pre-existing physical health condition | 42.9 | 57.1 |  |
| Mental Health |  |  | χ^2^ _(1)_ = 7.361, p = .007 |
| Pre-existing mental health condition | 47.4 | 52.6 |  |
| No pre-existing mental health condition | 40.1 | 59.9 |  |
| Wave 1 (month 1) Measures | M (SD) | M (SD) |  |
| Worries |  |  |  |
| About quarantine/self-isolation | 2.40 (1.15) | 2.37 (1.08) | T_(1388.270)_ = -.547, p = .588 |
| About being infected | 3.08 (1.18) | 3.11 (1.15) | T_(1601)_ = .384, p = .701 |
| About infecting others | 3.51 (1.21) | 3.54 (1.14) | T_(1601)_ = .558, p = .577 |
| About being stigmatised | 1.91 (1.19) | 1.78 (1.11) | T_(1601)_ = -2.114, p = .035 |
| About financial implications | 3.37 (1.34) | 3.38 (1.30) | T_(1601)_ = .216, p = .829 |
| About food shortages | 2.82 (1.22) | 2.63 (1.14) | T_(1601)_ = -3.353, p <.001 |
| About UK government ability | 3.44 (1.18) | 3.34 (1.18) | T_(1601)_ = -1.730, p = .084 |
| About Health service ability to cope | 3.93 (1.08) | 2081 (1.03) | T_(1601)_ = -.288, p = .773 |
| About impact of border closures | 2.27 (1.27) | 2.21 (1.22) | T_(1601)_ = -.941, p = .347 |
| Loneliness | 5.42 (1.96) | 5.28 (0.93) | T_(1605)_ = -1.439, p = .150 |
| Social Support | 21.23(6.01) | 21.92 (5.68) | T_(1605)_ = 2.356, p = .019 |
| Anxiety | 7.67 (6.20) | 7.00 (5.53) | T_(1402.478)_ = -2.189, p = .027 |
| Depression | 8.35 (6.72) | 7.37 (6.30) | T_(1390.031)_ = -2.957, p = .003 |
| Meaning in Life |  |  |  |
| Presence | 17.19 (5.48) | 17.34 (5.49) | T_(1590)_ = .589, p = .570 |
| Search | 20.02 (7.70) | 20.94 (7.71) | T_(1590)_ = -2.773, p = .006 |
